# PLGA‐Encapsulated Cross‐Linked GALC Aggregates as a Hierarchical Nanostructured Platform for Enzyme Stabilization and Delivery

**DOI:** 10.1155/ijbm/8346433

**Published:** 2026-10-18

**Authors:** Laura Colagiorgio, Mariacristina Gagliardi, Ambra Del Grosso, Sara Carpi, Miriam De Sarlo, Marco Cecchini

**Affiliations:** ^1^ Istituto Nanoscienze – CNR, NEST-SNS Piazza San Silvestro 12 56126 Pisa Italy; ^2^ Interdisciplinary Research Center “Health Science” Scuola Superiore Sant’Anna Piazza Martiri Della Libertà, 33 56127 Pisa Italy sssup.it

**Keywords:** cross-linked enzyme aggregates, hierarchical nanostructures, PLGA nanoparticles, polymer–enzyme nanocomposites

## Abstract

Enzyme aggregation and polymer encapsulation are complementary strategies used to overcome the intrinsic instability and limited bioavailability of hydrophilic enzymes in medical uses. In this work, we report a systematic study of polymer‐encapsulated cross‐linked enzyme aggregates (pe‐CLEAs) as a hierarchical nanostructured platform for the stabilization and delivery of galactosylceramidase (GALC), a lysosomal enzyme used in the enzyme replacement therapy for Krabbe disease.

GALC‐CLEAs were produced via cross‐linking and controlled precipitation, yielding stable aggregates with small hydrodynamic diameters (40–66 nm) and narrow polydispersity index (PDI < 0.1). Scaling up the synthesis by 50‐fold markedly enhanced total enzymatic output, reaching 1225.4 ± 213.2 U·mL^−1^. GALC‐CLEAs were subsequently encapsulated within PLGA nanoparticles. Systematic screening of enzyme‐to‐polymer ratios revealed an optimal formulation at 75 μg GALC per mg PLGA, displaying a maximum specific activity of 6.0 ± 1.3 U·mg^−1^.

Colloidal stability and retention assays showed that pe‐CLEAs preserved full catalytic activity and retained enzyme confinement over 30 days at 4°C. At 37°C, pe‐CLEAs exhibited time‐dependent size dynamics while retaining substantial activity. In vitro evaluations in primary fibroblasts derived from Twitcher mice (a Krabbe disease model) demonstrated complete cytocompatibility up to 5.0 mg·mL^−1^ and robust intracellular recovery of GALC activity across tested doses (0.5–5.0 mg·mL^−1^), outperforming equivalent doses of native enzyme.

This work provides a comprehensive physicochemical and functional characterization of pe‐CLEAs, elucidating how hierarchical aggregation and polymer encapsulation can synergistically preserve stability and bioactivity. The design principles outlined here support the rational development of polymer–enzyme nanocarriers for enzyme replacement therapy and related biomedical applications.

## 1. Introduction

Enzymes are highly efficient biocatalysts that play a central role in a wide range of biological and chemical processes owing to their exceptional specificity and selectivity [1]. Their applications span numerous industrial sectors, including food processing, textiles, paper manufacturing, and cosmetics [2–6]. In the biomedical field, enzymes are key components of high‐sensitivity biosensors, advanced diagnostic platforms, and therapeutic strategies [7–12].

Despite these advantages, the practical use of soluble enzymes is often limited by their poor operational stability [13]. Exposure to harsh conditions, such as elevated temperatures, extreme pH values, or organic solvents, frequently leads to denaturation and irreversible loss of catalytic activity [14].

These limitations have stimulated the development of alternative strategies based on controlled protein aggregation and self‐assembly, which emulate natural mechanisms employed by biological systems to preserve structure and function under adverse conditions.

Protein aggregation represents a fundamental organizational principle in living systems, enabling the formation of hierarchical architectures essential for cellular structure and function [15, 16]. In natural processes, including amyloid fibril formation and phase‐separated condensates [17], proteins self‐assemble into ordered aggregates that support stress responses, signaling pathways, and material‐like properties in biofilms [18]. Biomimetic materials exploit these protein assemblies to generate robust nanostructures with enhanced mechanical stability and catalytic performance [19, 20].

Inspired by these paradigms, carrier‐free enzyme immobilization strategies have been developed to harness aggregation for practical biocatalysis [21].

Among them, cross‐linked enzyme aggregates (CLEAs) have emerged as a versatile and support‐free immobilization platform. The preparation of CLEAs involves a two‐step process comprising enzyme precipitation from aqueous solution followed by chemical cross‐linking [22]. This method integrates purification and immobilization into a single operation, yielding permanently insoluble, stable, and recyclable biocatalysts with high volumetric productivity and without the need for expensive carrier materials.

Such systems exemplify aggregation‐induced stabilization [23, 24], in which precipitation combined with covalent cross‐linking generates insoluble enzyme assemblies that often outperform their soluble counterparts, particularly in terms of catalytic activity and operational stability [25, 26].

Recent studies highlight the broad applicability of this approach across different fields, ranging from industrial biocatalysis to emerging biomedical applications. For instance, nanomagnetic combi‐CLEAs incorporating viscozyme, alcalase, and flavourzyme have been developed for the simultaneous extraction of oils and proteins, showing improved catalytic efficiency together with facile magnetic recovery [27]. In a related example, *Penicillium notatum* lipase CLEAs cross‐linked with ethylene glycol‐bis (succinic acid N‐hydroxysuccinimide) achieved hydrolytic and esterification activities of 52% and 64%, respectively. Optimal operating conditions shifted to pH 9.0 and 42°C, while the immobilized system retained 71% of its initial activity after 10 reuse cycles, in contrast to the rapid deactivation observed for the free enzyme [28]. Furthermore, tyrosinase CLEAs exhibited a half‐life of 327 h in *tert*‐butanol at 40°C, compared with only 5.5 h in aqueous media, demonstrating markedly enhanced solvent tolerance attributed to multipoint covalent stabilization [29].

In the biomedical domain, enzymes and CLEAs are increasingly investigated for targeted delivery systems and enzyme replacement therapy (ERT) [30–32]. However, the efficient administration of functional enzymes remains challenging because of rapid clearance from the bloodstream, potential immunogenicity, and the presence of biological barriers [9].

Polymer–enzyme hybrids have emerged as advanced hierarchical systems in which enzyme aggregates are embedded within polymeric matrices to form composite nanostructures [33–35]. The interaction between the polymer scaffold and the enzyme assembly regulates release kinetics, provides protection against proteolytic degradation, and enhances targeting efficiency [36]. These hybrid materials exhibit synergistic behavior by combining polymer biodegradability with enzymatic activity and stability [37, 38].

For example, *β*‐glucuronidase encapsulated in PLGA nanoparticles by a double‐emulsion method using Tween® 80 retained 80% of its initial activity after encapsulation, compared with 40% for the free enzyme, and enabled sustained release over 6 days while preserving lysosomal targeting due to stabilization at aqueous interfaces [39]. Similarly, brain‐targeted PLGA nanoparticles loaded with arylsulfatase A enzyme aggregates achieved a threefold increase in lysosomal delivery in glial cells relative to the free enzyme. In this case, the PLGA matrix provided protection against blood–brain barrier proteases and enabled prolonged galactosylceramide hydrolysis [40].

Despite these advances, systematic investigations addressing the relationship between aggregation, size, and enzymatic activity in CLEAs remain scarce [41]. Moreover, scalability issues persist, including limited reproducibility in large‐volume precipitation and nonuniform cross‐linking efficiency [42]. These limitations highlight the need for more controlled and reproducible fabrication strategies to enable translational applications [43].

While the encapsulation of galactosylceramidase (GALC)‐CLEAs into polymeric nanoparticles has been previously demonstrated, including in our earlier work [40, 44], such studies have primarily focused on *proof-of-concept* applications, particularly in terms of in vivo protein delivery platform. A systematic understanding of how the parameters governing CLEAs formation and encapsulation influence the physicochemical and functional properties of the final system remains limited. Some key aspects, such as the relationship between enzyme‐cross‐linker ratio, enzyme loading, aggregate size, and catalytic activity, have not yet been comprehensively investigated. This lack of mechanistic and optimization studies hampers the rational design of aggregation‐based enzyme delivery systems for translational applications.

In this work, a hierarchical system based on CLEAs encapsulated within polymeric nanoparticles was developed and systematically characterized by tuning composition ratios and preparation parameters. Building on a previously reported protocol [44], we optimized and scaled up the production of CLEAs of GALC, and PLGA‐based nanoparticles obtained by nanoprecipitation and loaded with CLEAs.

The study first focused on improving the preparation procedures to increase nanoparticle yield and reproducibility while preserving enzymatic activity. The productions of both CLEAs and polymer‐encapsulated cross‐linked enzyme aggregates (pe‐CLEAs) were expanded to obtain a nanovector with maximized enzymatic performance, while monitoring variations in hydrodynamic diameter. The stability of particle size and enzymatic activity was then evaluated over time under different experimental and storage conditions.

Finally, the biological performance of the optimized nanostructures was investigated in vitro by assessing their biocompatibility and their ability to restore enzymatic activity in primary fibroblast cultures derived from Twitcher (TWI) mice, thus providing a proof of concept for their potential biomedical application. The experimental workflow proposed is schematized in Figure 1.

**FIGURE 1:**
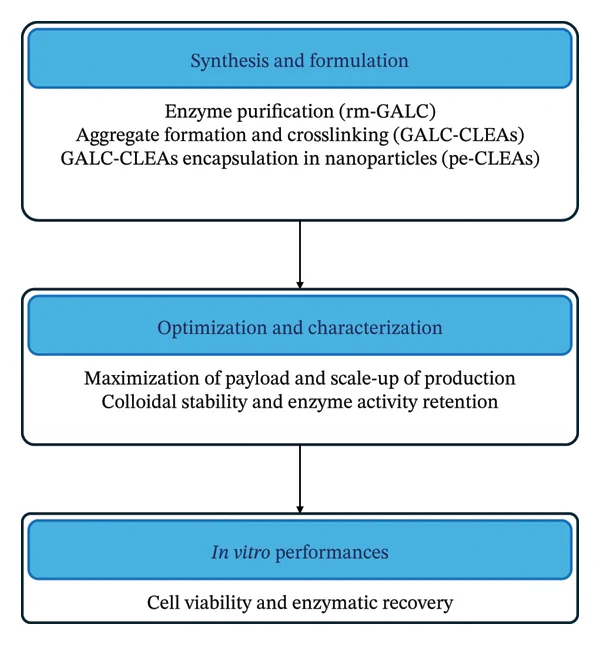
Experimental workflow of the study: Schematic representation illustrating the three main phases of the project: 1. Purification of rm‐GALC, formation of cross‐linked enzyme aggregates (GALC‐CLEAs), and encapsulation into functionalized PLGA nanoparticles (pe‐CLEAs); 2. Physicochemical optimization, scale‐up, and storage/stability assays; 3. In vitro validation in primary Twitcher fibroblasts, evaluating cell viability and enzyme activity recovery.

## 2. Materials and Methods

### 2.1. Reagents

Unless otherwise specified, all reagents were purchased from Sigma‐Aldrich and used as received.

Poly(D,L‐lactide‐*co*‐glycolide) carboxyl terminated (PLGA, Resomer RG 752H, *M*_*w*_ 4000–15000 Da, LA/GA molar ratio 75/25) was used as polymer in pe‐CLEAs preparation. Poly(D,L‐lactide‐*co*‐glycolide) carboxyl terminated (PLGA, Resomer RG 502H, *M*_*w*_ 7000–17000 Da, LA/GA molar ratio 50/50) was used to prepare the Angiopep‐2‐conjugated copolymer. Angiopep‐2 (TFFYGGSRGKRNNFKTEEYGC, *M*_*w*_ 2350 Da, purity > 95%, Biotek), N‐(3‐dimetilaminopropil)‐N′‐ethylcarbodiimide (EDC, crystalline), N‐hydroxysuccinimide (NHS, purity degree > 98%), N‐(2‐aminoethyl)maleimide trifluoroacetate (MAL, purity degree > 98%), and N,N‐dimethylformamide (DMF, HPLC grade) were used for PLGA functionalization. Sodium cholate (*M*_*w*_ 430.55 Da, purity degree 95%) was used as a surfactant for nanoparticle preparation. Trehalose (biochemistry degree) was used as cryoprotectant agent in the nanoparticle storage solution.

MES buffer (2‐morpholinoethanesulphonic acid), sodium chloride, glycerol, 4‐methylumbelliferyl‐β‐D‐galactopyranoside (4‐MU‐βGal), binding buffer (sodium phosphate monobasic 20 mM, NaCl 0.5 M, imidazole 25 mM; pH 7.4), wash buffer (20 mM sodium phosphate monobasic, 0.5 M NaCl, 25 mM imidazole; pH 7.4), elution buffer (20 mM sodium phosphate monobasic, 0.5 M NaCl, 500 mM imidazole; pH 7.4), storage buffer (MES 25 mM, sodium chloride 150 mM, glycerol 20% v/v; pH 5.5), phosphate‐buffered saline (PBS, 140 mM NaCl, 10 mM phosphate buffer and 3 mM KCl, pH 7.4 at 25°C), RIPA buffer (R0278‐50ML) containing a protease and phosphatase inhibitors cocktail (cOmplete [4693116001] and PhosSTOP [4906845001]; Roche Diagnostics), zeocin (R25001; Thermo Fisher Scientific), proteinase K digestion (spinNAker Universal Genomic DNA Mini Kit, Euroclone), Dulbecco’s modified Eagle medium (DMEM), fetal calf serum (FCS, 26010074; Thermo Fisher Scientific), 4 mM L‐glutamine (25030081; Thermo Fisher Scientific), 1% penicillin/streptomycin (15140122; Thermo Fisher Scientific), Pierce BCA Protein Assay (Micro BCA Protein Assay Kit, Thermo Fisher Scientific), 6‐hexadecanoylamino‐4‐methylumbelliferyl‐β‐D‐galactopyranoside (HMU‐βGal; EH05989, Deltek S.r.l., Pozzuoli [NA], Italy), and Ni Sepharose Excel resin (17‐3712‐03, GE Healthcare Life Sciences) were used for rm‐GALC purification protocol and in cell culture tests.

Ninhydrin (ACS grade), ethylene glycol (anhydrous, > 99.8%), tin (II) chloride (reagent grade, 98%), and hydrochloric acid (HCl, 37%, ACS reagent) were used for protein quantification assay.

The enzyme GALC, produced and purified in‐house, glutaraldehyde (25% water solution, *M*_*w*_ 100.12 Da, purity degree > 98%), acetone (ACS grade), and distilled water were used in GALC‐CLEAs preparation.

Heterozygous TWI mice (GALC^±^ C57BL/6J; Jackson Laboratory, Bar Harbor, ME, USA) were kindly provided by Dr. A. Biffi and Dr. A. Gritti (San Raffaele Telethon Institute for Gene Therapy, Milan, Italy).

### 2.2. rm‐GALC Purification and GALC‐CLEAs Synthesis

HEK293T cells stably expressing and secreting His6‐tagged murine wild‐type (WT) galactosylceramidase (GALC), kindly provided by J. Deane (University of Cambridge) [45], were maintained in selective medium containing 400 μM zeocin and expanded in roller bottles with an inner surface area of 1450 cm2 (37°C, 5% CO2, 0.7 rpm).

The enzyme was routinely purified from the conditioned culture medium by nickel affinity chromatography. Briefly, the culture medium was centrifuged at 1200–1300 rpm for 5 min, and the supernatant was filtered through a 0.45‐μm membrane filter. The clarified medium was then incubated overnight at 4°C under gentle agitation with binding buffer.

After 24 h, the solution was loaded onto the affinity column and washed six times with wash buffer. Elution was subsequently performed using one column volume of elution buffer collected in tubes equipped with a 10‐kDa molecular weight cutoff filter. The eluate was concentrated by three successive centrifugation steps at 4°C and 4000 rpm for 50 min, each followed by the addition of storage buffer.

The protein concentration of the purified recombinant murine GALC (rm‐GALC) was determined using the Pierce BCA Protein Assay at the end of the purification procedure. The purified enzyme in storage buffer was stored at −20°C until further use [32].

To optimize the formation of GALC‐CLEAs, the ratio between enzyme and cross‐linking agent was systematically varied. GALC‐CLEAs were prepared by precipitating rm‐GALC in acetone, followed by cross‐linking with glutaraldehyde. To do this, 200 μL of rm‐GALC solution (0.75 μg·µL^−1^ solubilized in the storage buffer) was diluted in 600 μL of acetone. Subsequently, glutaraldehyde was added under stirring. Different GALC‐to‐glutaraldehyde ratios were obtained by adjusting the amount of glutaraldehyde added to the enzyme solution, while keeping the total reaction volume constant. The tested ratios ranged from 0.07 to 0.60 μg·µg^−1^ (GALC/glutaraldehyde). The reaction was carried out on ice under a vented hood for 60 min. Following incubation, the mixture was centrifuged at 13,300 rpm for 5 min, and the supernatant was discarded. The resulting pellet, containing GALC‐CLEAs, was washed with 1 mL of water and centrifuged again under the same conditions. The final pellet was resuspended in 1 mL of acetone and stored at −20°C until further use. After this screening, we selected one GALC‐to‐glutaraldehyde ratio.

To improve the synthesis yield of GALC‐CLEAs, the original protocol was modified by increasing the enzyme amount (Table 1). Specifically, 300 μg of rm‐GALC (10‐fold higher than the baseline protocol) and 1500 μg (50‐fold higher) were used in separate reactions. All syntheses were performed in triplicate to assess procedural reproducibility.

**TABLE 1:** Nomenclature of recipes used for CLEAs synthesis, amounts and volumes of reactants used in the syntheses.

Recipe nomenclature	GALC amount (µg)	GALC solution volume (mL)	Acetone volume (mL)	Glutaraldehyde volume (mL)
1x	30	0.2	0.6	0.002
10x	300	2	6	0.02
50x	1500	10	30	0.1

### 2.3. Preparation of PLGA‐Ang and pe‐CLEAs

The PLGA copolymer had been previously functionalized with Angiopep‐2 according to Galliani et al. [44]. Briefly, PLGA functionalization with Angiopep‐2 was carried out through a two‐step procedure involving maleimide activation followed by peptide conjugation. In the first step, carboxyl‐terminated PLGA (500 mg, corresponding to 25 μmol) was dissolved in acetone. EDC (240 mg, 250 μmol) and NHS (144 mg, 250 μmol) were added along with 20 μL of triethylamine (TEA). The reaction mixture was stirred at room temperature for 1 h to activate the carboxylic groups of the PLGA end groups. Subsequently, MAL (63.5 mg, 50 μmol) was added, and the reaction was allowed to proceed under stirring at room temperature for 4 h. Upon completion, the solvent was removed under reduced pressure, and the resulting polymer was washed three times with methanol and then dried under vacuum to obtain maleimide‐functionalized PLGA (PLGA‐Mal). The product was stored at 4°C until further use. In the second step, PLGA‐Mal (10 μmol) was dissolved in DMF and reacted with Angiopep‐2 peptide (11.2 μmol) under stirring for 24 h at room temperature. After completion of the reaction, the solvent was removed under vacuum and the resulting polymer was washed three times with methanol to remove unreacted peptide, followed by vacuum drying. The final Angiopep‐2‐functionalized polymer (PLGA‐Ang) was stored at 4°C in a sealed glass vial until use. FT‐IR, ^1^H‐NMR, and DOSY‐NMR analyses were performed to confirm the successful conjugation.

For the preparation of pe‐CLEAs, 8 mg of PLGA and 2 mg of PLGA functionalized with Angiopep‐2 peptide were dissolved in 200 μL of acetone. Here, 200 μL of a preformed CLEAs suspension was added. The mixture was added dropwise to 2.4 mL of 2% (w/v) sodium cholate solution in water under vigorous shaking. After 1 h, the resulting nanoparticles were collected by centrifugation at 13,300 rpm for 5 min, the supernatant was discarded, and the pellet was washed with 1 mL of water. The final pe‐CLEAs were resuspended in 50 μL of trehalose solution (100 mg mL^−1^) and stored at −20°C until further use.

To optimize the nanoparticle preparation, the protocol was modified by increasing the amount of GALC‐CLEAs while keeping the PLGA content constant. Six different formulations were prepared in triplicate, corresponding to 3, 30, 75, 150, 225, and 300 μg of GALC per mg of PLGA. As a control, empty nanoparticles lacking GALC‐CLEAs were synthesized using 10 mg of commercial PLGA. Ctrl nanoparticles were obtained by dissolving 10 mg of commercial PLGA in 400 μL of acetone, then the solution was added dropwise to 2.4 mL of 2% (w/v) sodium cholate solution in water under vigorous shaking. After 1 h, the resulting nanoparticles were treated with the same procedure described for pe‐CLEAs.

### 2.4. Physicochemical Characterization

The chemical functionalization of PLGA with Angiopep‐2 was confirmed by proton nuclear magnetic resonance spectroscopy (^1^H‐NMR) and diffusion‐ordered spectroscopy (DOSY‐NMR). The corresponding unmodified PLGA was analyzed as a reference control.

For FT‐IR analysis, polymer samples were measured in their form, with no preliminary preparation. For ^1^H‐NMR analysis, polymer samples were dissolved in DMSO‐d_6_ at a concentration of 2 mg·mL^−1^, and spectra were recorded under standard conditions.

The enzymatic activity of rm‐GALC, GALC‐CLEAs, and pe‐CLEAs was determined using the fluorogenic substrate 4‐MU‐βGal. In this assay, 1 μL of each sample was mixed with 24 μL of assay buffer (50 mM sodium citrate, 125 mM sodium chloride, 0.5% Triton X‐100, pH 4.5) and 25 μL of 4‐MU‐βGal substrate solution (1 mM). The reaction mixture was incubated at 37°C for 2 h under mild stirring (300 rpm). The reaction was terminated by adding 150 μL of stop solution (0.5 M glycine, 0.3 M sodium hydroxide, pH 10.0). Samples were transferred to a 96‐well microplate, and the fluorescence intensity was measured using a GloMax Discover Microplate Reader (Promega).

The protein content of CLEAs was determined using a ninhydrin assay. Briefly, 10 μL of CLEAs suspension in acetone were subjected to acid hydrolysis by incubation overnight at 95°C in 200 μL of 6 M HCl, followed by vacuum drying. The resulting residue was resuspended in deionized water, and 10 μL of each sample was mixed with 110 μL of ninhydrin reagent (ninhydrin [20 mg·mL^−1^] and stannous chloride [2 mg·mL^−1^] in 75% ethylene glycol prepared in 4 N acetate buffer [pH 5.5]), heated at 95°C for 20 min, then cooled at room temperature, and transferred to a 96‐well microplate. Absorbance was measured at 560 nm.

Enzymatic activity in GALC and GALC‐CLEAs was expressed as units per microgram of enzyme (U μg^−1^) while in units per milligram of polymer (U mg^−1^) in pe‐CLEAs and calculated by comparison with a standard 4‐methylumbelliferone calibration curve.

The size of GALC‐CLEAs and pe‐CLEAs was determined by dynamic light scattering (DLS) using a Zetasizer instrument (Malvern Panalytical, Malvern, UK). For measurements on GALC‐CLEAs by varying the GALC‐to‐glutaraldehyde ratio, samples were prepared by diluting 10 μL of GALC‐CLEAs suspension in 100 μL of acetone. The resulting solution was transferred to a quartz cuvette for the analysis. For measurements on GALC‐CLEAs by varying the starting amount of GALC in the recipe, samples were prepared by diluting 10 μL of GALC‐CLEAs suspension in 400 μL of deionized water. The resulting solution was transferred to a quartz cuvette for the analysis. For measurements on pe‐CLEAs, samples were prepared by diluting 5 μL of nanoparticle suspension in 400 μL of deionized water and then loaded into the measurement cuvette. Size measurements were performed in triplicate for all formulations immediately after preparation (scattering angle = 90°, thermostatted at 25°C).

A stability study was performed on the pe‐CLEAs formulation containing 75 μg GALC per mg PLGA to evaluate changes in particle size and enzymatic activity over time. For such studies, both particle size and enzyme activity were monitored over a period of 30 days, at 4°C and at 37°C. Nanoparticles were stored at the fixed temperature in physiological solution. At predetermined time points, samples were collected and analyzed by DLS and enzymatic activity assays as described above.

An additional study to evaluate CLEAs retention was performed. Samples were prepared as described for stability tests and performed over a period of 6 days. At predetermined times, samples were collected and centrifuged (13,300 rpm for 5 min). The enzyme activity was measured both in recovered pellets and in the supernatants.

All tests were performed in triplicate.

### 2.5. Primary Cell Cultures and In Vitro Treatments

Heterozygous TWI mice GALC^±^ C57BL/6J, a murine model of GALC deficiency and Krabbe disease, were used as breeding pairs to generate homozygous (GALC^−/−^, HOM) and wild‐type (GALC^+^/^+^, WT) mice. The animals were maintained under standard housing conditions in accordance with protocols and ethical guidelines approved by the Italian Ministry of Health (permit no. 535/2018‐PR; starting date: 9 July 2018).

Genotyping was performed by extracting genomic DNA from tail biopsies following proteinase K digestion and applying a real‐time PCR allelic discrimination assay [46].

Adult fibroblast cultures were established from WT and TWI mouse ears [47]. Cells were cultured in high‐glucose DMEM supplemented with 10% heat‐inactivated FCS, 4 mM L‐glutamine, and 1% penicillin/streptomycin.

For treatments, WT and HOM fibroblasts were seeded in 96‐well plates at a density of 12,500 cells/well. After 24 h, the cells were exposed for 4 h to different concentrations (5.0, 2.5, 1.0, and 0.5 mg·mL^−1^) of pe‐CLEAs containing 75 μg GALC per mg PLGA, diluted in complete culture medium. Cell viability was evaluated using the RealTime‐Glo™ MT Cell Viability Assay (Promega) following PBS washing, and luminescence was recorded with a GloMax Discover Microplate Reader (Promega). Results were expressed as percentage relative to untreated WT controls. Each condition was tested in duplicate within each experiment, with at least three independent biological replicates.

For intracellular rm‐GALC activity measurements, culture medium was removed after treatment, and cells were washed three times with PBS before lysis on ice with 35 μL of RIPA buffer containing protease and phosphatase inhibitors. Cell lysates were sonicated (4 s at 12‐μm intensity) and centrifuged at 13,000 rpm for 5 min at 4°C. Protein concentration in the supernatants was determined using the Pierce BCA Protein Assay. Enzymatic activity was quantified using HMU‐βGal [48]. Aliquots of 10 μL of cell lysates were incubated with 20 μL of 50 μM HMU‐βGal for 17 h at 37°C. The reaction was stopped, and the fluorescence of the released 4‐MU was measured using a GloMax plate reader. GALC activity was expressed as nanomoles of product per milligram of total protein over 17 h and calculated by comparison with a standard curve. Statistical analysis was performed using Prism software (GraphPad Software, San Diego, CA; RRID: SCR_002798) by one‐way ANOVA followed by Sidak’s multiple‐comparisons test.

A schematic summary of the present work is reported in Figure 2.

**FIGURE 2:**
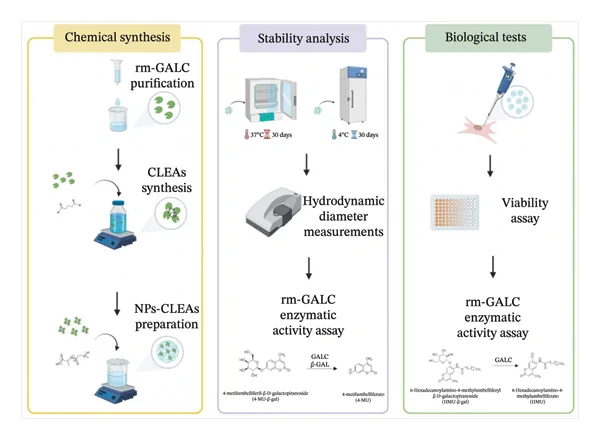
Schematic representation of the experimental workflow for rm‐GALC nanoparticle fabrication, characterization, and biological evaluation. The process comprises three main stages. Chemical synthesis (left): Purified recombinant murine GALC (rm‐GALC) was cross‐linked with glutaraldehyde to form CLEAs, subsequently encapsulated into PLGA‐based nanoparticles (pe‐CLEAs). Stability analysis (center): Formulations were stored at 4°C and 37°C for up to 30 days, monitoring hydrodynamic diameter and enzymatic activity using 4‐MU‐βGal. Biological testing (right): Cytocompatibility was assessed by cell viability assays, and intracellular enzymatic activity was quantified using HMU‐βGal to evaluate functional delivery.

## 3. Results

This work presents the systematic investigation of CLEAs formation and their encapsulation into polymeric nanoparticles. In particular, the effects of enzyme loading and formulation parameters on particle size, enzymatic activity, stability, and biological performance are analyzed.

### 3.1. PLGA‐Ang, GALC‐CLEAs, and pe‐CLEAs Characterization

The FT‐IR and ^1^H‐NMR characterization confirmed the successful functionalization of the commercial polymer with the peptide Angiopep‐2 (Supporting Information, Figures S1.1–S1.9).

The FT‐IR spectrum of the commercial PLGA copolymer exhibited an absorption peak observed at 1750–1760 cm^−1^ (carbonyl stretching vibration of the ester groups), bands in the range between 2950 and 3000 cm^−1^ (aliphatic C–H stretching vibrations of –CH_3_ and –CH_2_– groups), and bands in the range between 1080 and 1180 cm^−1^ region (stretching of ether and ester bonds). Additional signals existed in Angiopep‐2 around 3300 cm^−1^ (stretching of hydroxyl group), at 1650–1690 cm^−1^ and 1500–1600 cm^−1^ (stretching vibration of the amide bond and benzene ring).

^1^H‐NMR: (300 MHz, DMSO‐d6): *δ*/ppm 5.2 (–OCH(CH_3_)COO–), 4.9 (–OCH_2_COO–), 1.5 (–OCH(CH_3_)COO–); additional signals at 6.7, 6.9,7.2, 8.0, and 8.1 appeared in the Angiopep‐functionalized polymer [49].

The hydrodynamic diameter of GALC‐CLEAs slightly varied with the GALC‐to‐glutaraldehyde ratio, ranging from approximately 40 to 66 nm (Figure 3a). The smallest sizes were observed at ratios of 0.15 and 0.20 mg·mg^−1^, while larger diameters were measured at both higher and lower ratios. The values of PDI ranged from 0.03 to 0.1 (Figure 3b), indicating very narrow size dispersions, as also shown by cumulative distributions (Figure 3d–f). The specific enzyme activity ranged between 0.29 and 0.66 U·μg^−1^ of GALC without any specific trend (Figure 3c). Enzyme activity (U·mL^−1^) and protein content (μg·mL^−1^) of GALC‐CLEAs solution are summarized in Table 2. The average yield of GALC‐CLEAs production, calculated on the protein content measured, was 0.52 ± 0.13 (SE) with respect to the theoretical GALC concentration in the production (150 μg·mL^−1^).

**FIGURE 3:**
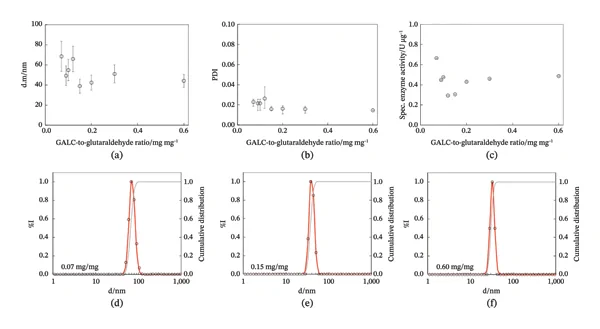
Optimization of the GALC‐to‐glutaraldehyde ratio in GALC‐CLEAs synthesis: (a) size (nm) and (b) polydispersity index (PDI) measured by DLS, (c) specific enzyme activity over the GALC protein content (U μg^−1^), (d–f) DLS traces obtained by measuring GALC‐CLEAs with different glutaraldehyde contents (o, experimental data; 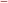, data fitting; —, cumulative distribution).

**TABLE 2:** Enzyme activity and protein content of GALC‐CLEAs obtained by varying the GALC‐to‐glutaraldehyde ratio, and enzyme activity and protein content of the native GALC solution.

GALC/glutaraldehyde (mg·mg^−1^)	Enzyme activity (U·mL^−1^)	Protein content (μg·mL^−1^)
0.07	34.2	70.3
0.09	33.7	73.4
0.10	35.3	82.2
0.12	35.7	117.4
0.15	21.0	71.7
0.20	34.0	71.7
0.30	33.2	74.0
0.60	38.4	57.9
GALC	35.5	238.2

At the end of this screening, we have selected the GALC‐CLEAs formulation with a GALC‐to‐glutaraldehyde ratio of 0.3 mg·mg^−1^.

The effect of GALC concentration on CLEA formation was assessed by scaling the initial formulation (theoretical GALC concentration: 150 μg·mL^−1^) by 10x and 50x.

Particle size analysis performed in aqueous medium revealed a marked increase in hydrodynamic diameter with increasing enzyme loading (Figure 4a). The mean size increased from approximately 600–700 nm for the 1x formulation to ∼800–900 nm for the 10x formulation, reaching ∼1800–2000 nm for the 50x formulation. These values are substantially higher than those previously measured in acetone, indicating a higher degree of aggregation under aqueous conditions.

**FIGURE 4:**
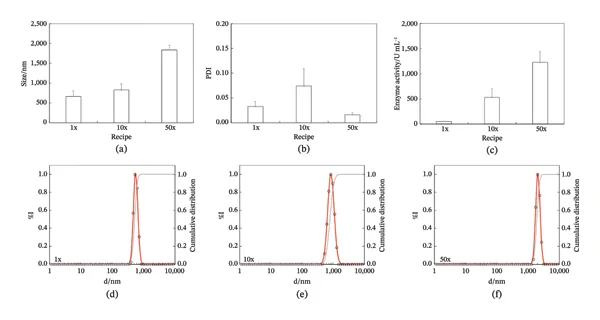
Characterization of GALC‐CLEAs obtained after the scale‐up of the recipe: (a) size (nm) and (b) polydispersity index (PDI) measured by DLS; (c) specific enzyme activity of resuspended CLEAs (U·mL^−1^); (d–f) DLS traces obtained by measuring GALC‐CLEAs with different recipes (o, experimental data; 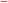, data fitting; —, cumulative distribution).

Despite the increase in size, all formulations exhibited relatively low polydispersity indices (PDIs < 0.1, Figure 4b), suggesting the formation of homogeneous populations of aggregates rather than broad or heterogeneous size distributions.

DLS traces showed unimodal size distributions for all samples (Figure 4d–f). The cumulative distributions displayed a single inflection point, and no secondary peaks or shoulders were observed, indicating the absence of large, irregular macroaggregates.

Enzymatic activity increased markedly with increasing initial GALC concentration (Figure 4c, Table 3). The 10x and 50x formulations exhibited a substantial enhancement in activity compared to the 1x sample, supporting the effectiveness of the scale‐up strategy.

**TABLE 3:** Measured enzyme activity in GALC‐CLEAs scale up by increasing the amount of GALC (μg) in the recipe (U·mL^−1^, Figure 3c).

Recipe	Enzyme activity ± SE (U·mL^−1^)
1x	48.0 ± 6.8
10x	529.7 ± 181.1
50x	1225.4 ± 213.2

Following the optimization of the initial GALC amount in the CLEAs synthesis protocol, the formulation exhibiting the highest enzymatic activity was selected, independently of the aggregation state of the CLEAs. This choice was based on the subsequent nanoparticle preparation process, which relies on acetone as a common solvent for both the polymer and CLEAs, ensuring their effective co‐dissolution. Among the tested conditions, the formulation identified as 50x was selected for further experiments.

Using these optimized GALC‐CLEAs, polymeric nanoparticles were prepared by varying the enzyme‐to‐polymer ratio in the range of 3–300 μg·mg^−1^. The resulting nanoparticles exhibited an overall increase in hydrodynamic diameter with increasing enzyme loading (Figure 5a), while the PDI remained relatively constant across all formulations (Figure 5b).

**FIGURE 5:**
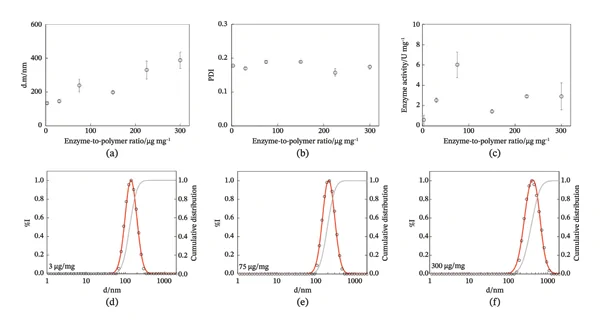
Characterization of NP‐CLEAs obtained by varying the ratio between GALC‐CLEAs and polymer: (a) size (nm) and (b) polydispersity index (PDI) measured by DLS; (c) specific enzyme activity with respect to the polymer amount (U·mg^−1^); (d–f) DLS traces obtained by measuring NP‐CLEAs with different GALC‐to‐polymer ratios (o, experimental data; 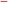, data fitting; —, cumulative distribution).

The corresponding DLS intensity distributions (Figure 5d–f) showed monomodal profiles without evidence of large aggregates, indicating that GALC‐CLEAs can be effectively co‐dissolved with the polymer in acetone without inducing secondary aggregation. A progressive shift in size distributions toward higher diameters was observed with increasing enzyme‐to‐polymer ratio.

In contrast, the specific enzymatic activity (Figure 5c, Table 4) did not follow a monotonic trend as a function of enzyme loading. Notably, a maximum in activity was observed for the formulation with an enzyme‐to‐polymer ratio of 75 μg·mg^−1^. Based on this result, this formulation was selected for subsequent studies.

**TABLE 4:** Measured enzyme activity in pe‐CLEAs optimized by increasing the enzyme‐to‐polymer ratio (U·mg^−1^, Figure 4c).

Enzyme‐to‐polymer ratio (μg·mg^−1^)	Enzyme activity ± SE (U·mg^−1^)
3	0.6 ± 0.4
30	2.5 ± 0.2
75	6.0 ± 1.3
150	1.4 ± 0.1
225	2.9 ± 0.1
300	2.9 ± 1.3

### 3.2. Functional Characterization of pe‐CLEAs

The colloidal stability and enzymatic performance of pe‐CLEAs were monitored over 30 days using the formulation containing 75 μg GALC per mg of PLGA. Empty polymeric nanoparticles (Ctrl) were analyzed in parallel and are reported as reference systems.

DLS measurements highlighted distinct stability profiles for Ctrl and pe‐CLEAs (Figure 6a,b). When stored at 4°C, Ctrl nanoparticles displayed highly stable hydrodynamic diameters throughout the entire monitoring period, with minimal dispersion over time. In contrast, pe‐CLEAs exhibited a broader size distribution already at early time points, indicating a lower degree of size homogeneity compared to Ctrl, although no abrupt size changes were detected at this temperature (Figure 6a).

**FIGURE 6:**
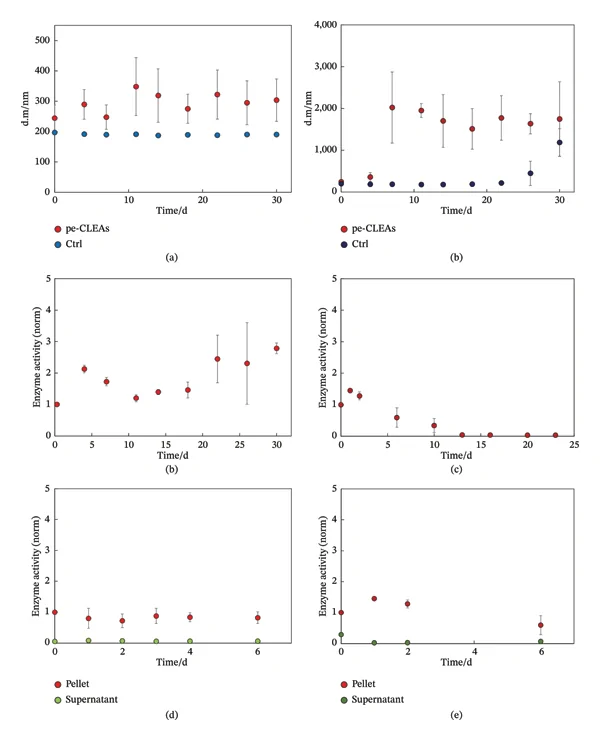
Characterization of pe‐CLEAs (formulation 75 μg GALC/mg PLGA): size stability with time at (a) 4°C and (b) 37°C and related enzyme activity normalized on the starting activity (day 0) at (c) 4°C and (d) 37°C; CLEAs retention test by evaluating the enzyme activity over time at (e) 4°C and (f) 37°C.

At 37°C, Ctrl nanoparticles showed a time‐dependent evolution of hydrodynamic diameter. Size values remained relatively stable during the initial storage period but progressively deviated after approximately 3 weeks, consistent with the onset of polymer degradation phenomena (Figure 6b). Conversely, pe‐CLEAs displayed a different behavior: particle size remained relatively stable only during the first 7 days of incubation, after which the measured diameters progressively increased and became more dispersed, indicating a loss of colloidal stability at prolonged exposure to physiological temperature (Figure 6b).

The enzymatic activity of pe‐CLEAs was also monitored at 4°C and 37°C (Figure 6c–d). When stored at 4°C, pe‐CLEAs showed no loss of activity over 30 days (Figure 6c). Normalized activity values remained consistently above the initial level throughout the monitoring period, with a moderate increase observed at later time points. Variability among replicates increased after day 20, although average activity values remained within the same order of magnitude. On the contrary, at 37°C, the enzymatic activity showed a mild increase at early times and then a progressive decline over time (Figure 6d).

Enzyme retention within pe‐CLEAs was further evaluated by measuring the activity associated with both the particulate fraction and the dilution medium over time (Figure 6e,f). At 4°C, values of the retained enzymatic activity by nanoparticles remained constant with time, indicating effective enzyme confinement within the polymeric matrix during storage. This behavior was confirmed by the very low enzyme activity measured in the storage medium (Figure 6e). At 37°C, retained activity exhibited a quite broader distribution and a certain activity was measured in the supernatant at early times (Figure 6f).

The biocompatibility of pe‐CLEAs was assessed in TWI fibroblasts by measuring cell viability after 4 h of exposure. Figure 7a depicts the viability of TWI and WT fibroblasts treated with pe‐CLEAs containing 75 μg GALC/mg PLGA at concentrations of 5, 2.5, 1, and 0.5 mg·mL^−1^. In both WT and HOM fibroblasts, cell viability remained comparable to the untreated control (CTRL) across all concentrations tested, with no statistically significant reduction observed.

**FIGURE 7:**
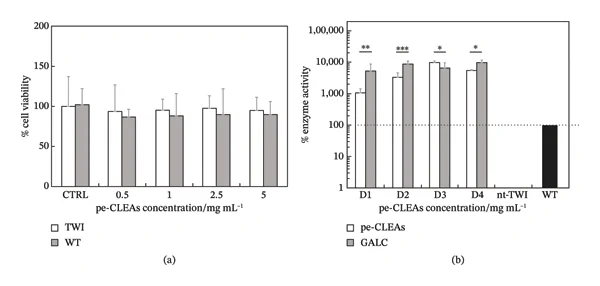
In vitro cell culture tests: (a) cell viability of TWI and WT fibroblasts measured after 4‐h treatment with pe‐CLEAs (75 μg GALC/mg PLGA) at the indicated concentrations (0.5, 1.0, 2.5, and 5.0 mg·mL^−1^), expressed as percent relative to untreated control (CTRL). (b) Enzymatic recovery experiments on TWI fibroblasts treated with increasing doses of pe‐CLEAs ranging from 0.5 to 5 mg·mL^−1^ (see Table 5), and with a corresponding activity given by free GALC, measured after 4‐h treatment; activity is expressed as percent normalized to WT levels, data points represent mean ± SD on a logarithmic scale (*n* = 4). ^∗∗∗^ *p* < 0.001, ^∗∗^ *p* < 0.01, ^∗^ *p* < 0.05 specific treatment with pe‐CLEAs versus GALC treatment, one‐way analysis of variance (ANOVA) (Sidak’s multiple‐comparisons test).

GALC enzymatic recovery in TWI fibroblasts was assessed following treatment with pe‐CLEAs or equivalent doses of native GALC. Enzyme activity in cell lysates was quantified (U·mg^−1^) and normalized to WT fibroblasts (referenced as full activity). Untreated TWI fibroblasts (nt‐TWI) displayed minimal activity, confirming the expected deficiency. Treatment with pe‐CLEAs across concentrations ranged from 0.5 to 5.0 mg·mL^−1^, corresponding to the enzyme activity reported in Table 5.

**TABLE 5:** Administered enzyme activity, by means of pe‐CLEAs or by free GALC, to cell cultures, and corresponding pe‐CLEAs concentration in the administered treatment.

Dose	Administered enzyme activity (U)	pe‐CLEAs concentration (mg·mL^−1^)
D1	70	0.5
D2	340	1.0
D3	850	2.5
D4	1700	5.0

In all cases, treated cells showed an enzyme activity higher than the one evaluated in WT cells (Figure 7b).

## 4. Discussion

This study was designed to systematically optimize the production of CLEAs of GALC and their subsequent encapsulation into polymeric nanoparticles, with the specific aim of understanding how each fabrication step influences the functional properties of the resulting hierarchical system. Compared to previous studies, including our earlier work [44], which primarily demonstrated proof‐of‐concept feasibility, the present work provides a systematic and quantitative investigation of the relationships between CLEAs formation parameters, nanoparticle composition, and functional performance. Rather than treating polymer encapsulation as a passive loading strategy, the present work investigates how enzyme aggregation, cross‐linking density, and enzyme‐to‐polymer ratio collectively determine size, stability, enzymatic performance, and biological activity.

The successful functionalization of the polymer with Angiopep‐2, confirmed by spectroscopic analysis, provided the integration of targeting functionality within the nanoparticle structure.

At the level of GALC‐CLEAs formation, protein quantification analysis provided an estimation of the cross‐linking yield and the enzyme activity after the chemical procedure, supporting the efficiency of the aggregation process. Moreover, modulation of enzyme concentration during the precipitation and cross‐linking step enabled the production of nanoscale aggregates with narrow size distributions and progressively increasing enzyme activity. The nearly linear relationship observed between enzyme loading and GALC‐CLEAs activity indicates that the aggregation process preserves the enzymatic activity while increasing overall catalytic output. This behavior is consistent with aggregation‐induced stabilization mechanisms, whereby multipoint covalent cross‐linking restricts conformational flexibility without impairing access to the catalytic sites [25, 41]. Importantly, the small hydrodynamic diameters and low polydispersity values obtained across the tested conditions demonstrate that the GALC‐CLEAs synthesis protocol is robust and reproducible.

Encapsulation of GALC‐CLEAs into PLGA‐based nanoparticles introduced an additional level of structural complexity, transforming the system into a hierarchical composite in which enzyme aggregates act as functional building blocks rather than as simple cargo. Systematic variation of the enzyme‐to‐polymer ratio revealed a clear trade‐off between particle size and enzymatic performance. While increasing GALC‐CLEAs loading led to larger nanoparticles, enzymatic activity exhibited a nonmonotonic trend, with a significant maximum at an intermediate loading (75 μg GALC per mg PLGA). This result highlights the importance of formulation optimization: excessive enzyme content likely increases mass‐transfer limitations or steric hindrance within the polymer matrix, whereas insufficient loading reduces overall catalytic output. This observation is in line with previous reports on CLEAs and immobilized enzyme systems, where excessive enzyme loading can lead to mass‐transfer limitations or steric hindrance, resulting in reduced catalytic efficiency despite increased enzyme content [21, 26].

The stability studies further demonstrate how the optimized pe‐CLEAs formulation behaves over time under different temperature conditions. At 4°C, pe‐CLEAs maintained relatively stable size distributions and preserved enzymatic activity over 30 days, confirming that the combination of enzyme aggregation and polymer encapsulation effectively protects the catalytic function during prolonged storage. Notably, normalized activity values measured on resuspended samples remained consistently at or above the initial level, hypothetically due to a gradual relaxation of the polymer–enzyme composite during storage. This characterization confirmed that long‐term storage does not compromise enzymatic performance. CLEAs retention tests confirmed that this behavior is not associated with enzyme release, as activity remained largely confined within the nanoparticle pellet and negligible activity was detected in the supernatant. Taken together, these findings indicate that the optimization of the pe‐CLEAs formulation ensures both physical retention of the enzyme and accessibility to the catalytic sites. This hierarchical organization is consistent with previously reported polymer–enzyme composite systems, in which enzyme aggregates act as structural building blocks that influence both physical and functional properties of the material [33, 34, 44].

At 37°C, pe‐CLEAs displayed a more dynamic behavior, characterized by earlier changes in particle size and a gradual decline in enzymatic activity. Importantly, the temporal evolution of activity did not directly mirror size changes, suggesting that structural rearrangements within the polymer–enzyme composite and thermal deactivation processes contribute differently to functional performance. While partial enzyme release was detected at early time points, most of the enzymatic activity remained associated with the particulate fraction, indicating that even under accelerated conditions, the hierarchical structure retains a significant degree of integrity. In this context, empty nanoparticles were used exclusively as polymeric references, confirming that the observed functional trends arise from the presence and organization of CLEAs within the polymer matrix rather than from the polymer component.

The functional relevance of the optimized pe‐CLEAs formulation was ultimately confirmed in primary fibroblasts derived from a murine model of Krabbe disease. pe‐CLEAs were well tolerated across the tested concentration range and enabled efficient intracellular recovery of GALC activity, surpassing the effect of equivalent doses of free enzyme. This outcome reflects the cumulative impact of the optimization strategy, in which controlled aggregation, stabilizing cross‐linking, and polymer encapsulation collectively enhance enzyme stability, availability, and cellular efficacy. These results further extend previous findings on polymer–enzyme nanocarriers for lysosomal enzyme delivery, highlighting the importance of controlled aggregation in achieving both stability and biological functionality [39, 40].

A limitation of the present study is the lack of direct structural characterization of GALC‐CLEAs, both as isolated aggregates and when embedded within the polymer matrix, at the molecular level. While the combination of DLS measurements and enzymatic activity assays provides robust evidence of aggregation and enzyme confinement, more advanced techniques could offer additional insight into the structural organization of the system. Complementary approaches such as microscopy or spectroscopic methods could help elucidate the spatial arrangement of GALC‐CLEAs within the polymer matrix and provide further information on their degree of confinement.

In addition, the biological evaluation performed in this work was limited to in vitro models. Nevertheless, a previous study from our group [40] demonstrated the applicability of a similar system in vivo and provided a more extensive biological characterization. Building on those findings, further long‐term in vivo studies will be required to fully validate the translational potential of the optimized pe‐CLEA platform.

Overall, this work demonstrates that the performance of polymer‐encapsulated CLEAs is dictated by the engineering of each hierarchical level, from enzyme aggregation to nanoparticle formulation. By systematically optimizing these parameters and correlating them with physicochemical and biological readouts, the present study establishes a rational framework for the design of aggregation‐based enzyme nanocarriers. Beyond the specific case of GALC, the principles identified here are broadly applicable to other therapeutic enzymes, providing a versatile strategy for improving stability and function in ERTs and related biomedical applications. In this context, the present study goes beyond proof‐of‐concept demonstrations by establishing quantitative structure–function relationships that enable the rational optimization of CLEAs‐based polymeric nanocarriers.

## 5. Conclusion

This study presents a systematic investigation of pe‐CLEAs as a hierarchical nanostructured platform for enzyme stabilization and delivery. By combining controlled enzyme aggregation with polymeric encapsulation, we demonstrate how structural organization across multiple length scales critically determines colloidal stability, enzymatic performance, and biological functionality.

Through the optimization of formulation parameters and an extensive physicochemical characterization, supported by complementary spectroscopic analyses confirming polymer functionalization, we show that pe‐CLEAs exhibit distinct size evolution and activity profiles compared to empty polymeric nanoparticles, particularly under physiologically relevant conditions. While polymer degradation governs the long‐term behavior of control nanoparticles, the presence of enzyme aggregates introduces additional structural dynamics that affect size dispersion without compromising enzyme confinement. Importantly, enzymatic activity remains largely retained within the polymeric matrix over extended time periods, highlighting the protective role of aggregation combined with encapsulation.

At the cellular level, pe‐CLEAs displayed excellent biocompatibility and enabled efficient recovery of GALC activity in fibroblasts derived from a murine model of Krabbe disease. These results indicate that the hierarchical architecture of pe‐CLEAs not only preserves enzymatic function but also supports effective intracellular delivery, overcoming key limitations associated with soluble enzyme formulations.

This work provides a mechanistic and engineering framework for the rational design of aggregation‐based polymer–enzyme composites. Beyond the specific case of GALC, the principles outlined here are broadly applicable to the development of stable and functional enzyme nanocarriers, opening new perspectives for ERT and related biomedical applications.

Future work will focus on a deeper structural characterization of the polymer–enzyme composite to better elucidate the spatial organization and confinement of GALC‐CLEAs within the polymer matrix. In parallel, further investigations will address intracellular trafficking mechanisms to clarify how the hierarchical structure influences cellular uptake and processing. In vivo studies will be expanded to evaluate long‐term therapeutic efficacy and biodistribution, building on previous in vivo evidence reported by our group. Finally, the design principles identified in this work will be extended to other therapeutic enzymes to assess the general applicability of the platform.

## Funding

This work was supported by European Leukodystrophy Association (ELA) International under the framework of the projects “nanoERT–Nanoparticle based Enzyme Replacement Therapy for the treatment of Krabbe disease: a pre‐clinical study in the Twitcher Mouse” (grant agreement no: G.A. ELA n. 2019–008I2AV1–NanoERT) and “Study of GPR65 as a new Krabbe disease drug target” (grant agreement no: ELA n. 2023‐006C4). Open access publishing was facilitated by Consiglio Nazionale delle Ricerche, as part of the Wiley–CRUI‐CARE agreement.

## Conflicts of Interest

The authors declare no conflicts of interest.

## Supporting Information

Additional supporting information can be found online in the Supporting Information section.

## Supporting information


**Supporting Information** The Supporting Information provides additional spectroscopic characterization of the Angiopep‐2‐functionalized PLGA copolymer. It includes FT‐IR spectra of commercial PLGA, maleimide‐functionalized PLGA (PLGA‐Mal), free Angiopep‐2, and Angiopep‐2‐conjugated PLGA (PLGA‐Ang) (Figure S1.1). ^1^H‐NMR spectra of PLGA, Angiopep‐2, and PLGA‐Ang, together with an expanded analysis of the 5.8–8.6 ppm region, are provided to support the successful peptide conjugation (Figures S1.2–S1.5). DOSY‐NMR spectra of PLGA, PLGA‐Mal, Angiopep‐2, and PLGA‐Ang are also included, confirming the covalent attachment of Angiopep‐2 to the polymer backbone through the co‐diffusion of peptide and PLGA resonances (Figures S1.6–S1.9).

## Data Availability

The datasets generated and/or analyzed during the current study are available on Zenodo with the following DOI: 10.5281/zenodo.21069028.
