# Supplementary material for: PLGA‐Encapsulated Cross‐Linked GALC Aggregates as a Hierarchical Nanostructured Platform for Enzyme Stabilization and Delivery
Source: Int J Biomater. 2026 Oct 18;2026:8346433. doi: 10.1155/ijbm/8346433 (PMC13678281; doi:10.1155/ijbm/8346433)
Supplement: Supplementary file 1 [file IJBM-2026-8346433-s001.docx]

**Supplementary information**

**FT-IR spectra**

As shown in S1.1, The chemical structure and successful conjugation steps were confirmed by FT-IR spectroscopy. Pristine PLGA exhibited characteristic absorption bands at 1750 cm^-1^ (-C=O ester stretching) and 1080-1180 cm^-1^ (-C-O- stretching). Following functionalization (PLGA-Mal), a shoulder at 1700-1710 cm^-1^ (-C=O imide) and a weak peak at 1600-1630 cm^-1^ assigned to the maleimide -C=C- double bond emerged. Free Angiopep-2 showed its signature peptide bands: Amide I at 1650 cm^-1^ (C=O stretch), Amide II at 1540 cm^-1^ (-N-H bending and -C-N- stretch), and a broad -N-H/-O-H stretching region around 3300 cm^-1^. In the PLGA-Angiopep conjugate spectrum, successful peptide attachment was confirmed by the co-existence of the PLGA ester peak (1750 cm^-1^ alongside the Amide I and II bands (1650 cm^-1^and 1540 cm^-1^). The disappearance of the maleimide -C=C- absorption band around 1600 cm^-1^ confirmed the reaction of the maleimide double bond with the cysteine thiol group of Angiopep-2.

S1.1. FT-IR spectra of commercial PLGA, maleimide-functionalized PLGA (PLGA-Mal), Angiopep-2 and Angiopep-2-conjugated PLGA (PLGA-Angiopep).

**^1^H-NMR spectra**

^1^H-NMR spectrum of commercial PLGA (S1.2) showed characteristic peaks at 1.5 ppm, 4.9 ppm, 5.2 ppm. Angiopep-2 (S1.3) showed characteristic signals for aromatic residues (Phe/Tyr, 6.6-7.4 ppm), amide protons (7.5-8.5 ppm), and aliphatic side chains (0.8–3.0 ppm). Angiopep-2-conjugated copolymer (S1.4) had additional peaks from benzene ring and amide bond between 6.5 ppm to 8.2 ppm.

The chemical structures and successful conjugation were further validated by analysing the spectra in the 5.8-8.7 ppm region (S1.5). The spectrum of pristine PLGA displayed a baseline with no resonances in this downfield region. For PLGA-maleimide, a sharp peak appeared at 7.0 ppm, corresponding to the olefinic protons (-CH=CH-) of the maleimide ring, accompanied by amide linker signals at 7.7-8.4 ppm. Free Angiopep-2 exhibited characteristic aromatic resonances from its phenylalanine and tyrosine residues at 7.1-7.3 ppm and 6.7 ppm, alongside broad backbone amide protons 7.3-8.5 ppm. In the PLGA-Angiopep conjugate, the complete disappearance of the maleimide double-bond peak at 7.0 ppm confirmed the reaction of the maleimide ring with the cysteine thiol group of Angiopep-2. Furthermore, the persistence of the peptide aromatic signals (7.2 ppm and 6.7 ppm) together with a distinct amide resonance at 8.0 ppm confirmed the successful covalent attachment of the peptide to the polymer.

S1.2. ^1^H-NMR spectrum of commercial PLGA.

S1.3. 1H-NMR spectrum of the peptide Angiopep-2.

S1.4. ^1^H-NMR spectrum of Angiopep-2-conjugated PLGA.

S1.5. ^1^H-NMR spectrum in the range between 5.8 ppm and 8.6 ppm of commercial PLGA (red), maleimide-functionalized PLGA (blue), Angiopep-2 (purple) and Angiopep-2-conjugated PLGA (green).

To further confirm covalent coupling, Diffusion-Ordered Spectroscopy (DOSY-NMR) was conducted to evaluate the self-diffusion coefficients (D) of the individual components and final conjugate.

Pristine PLGA exhibited a unified diffusion trace around 0.05 × 10^-9^ m^2^/s for all characteristic backbone resonances (δ = 1.5, 4.8, and 5.2 ppm). In the PLGA-maleimide spectrum, the maleimide olefinic resonance at 7.0 ppm displayed the exact same diffusion coefficient as the PLGA chain, confirming successful end-group functionalization. Free Angiopep-2 exhibited a distinctly higher self-diffusion coefficient, around 0.10 × 10^-9^ m^2^/s, consistent with its smaller hydrodynamic radius compared to the polymer. In the PLGA-Angiopep conjugate spectrum, the aromatic signals due to the peptide (7.2 ppm) shifted down in diffusion rate, aligning perfectly with the lower diffusion line of the PLGA backbone (D was found around 0.05 × 10^-9^ m^2^/s). The complete absence of residual peptide signals at D ~ 0.10 × 10^-9^ m^2^/s demonstrates the covalent conjugation between PLGA and Angiopep-2 rather than physical mixture, along with the high purity of the final product.

S1.6. DOSY-NMR spectrum of commercial PLGA.

S1.7. DOSY-NMR spectrum of maleimide-functionalized PLGA.

S1.8. DOSY-NMR spectrum of Angiopep-2.

S1.9. DOSY-NMR spectrum of Angiopep-conjugated PLGA.
